# Inhibition of miR-1193 leads to synthetic lethality in glioblastoma multiforme cells deficient of DNA-PKcs

**DOI:** 10.1038/s41419-020-02812-3

**Published:** 2020-07-30

**Authors:** Jing Zhang, Li Jing, Subee Tan, Er-Ming Zeng, Yingbo Lin, Lingfeng He, Zhigang Hu, Jianping Liu, Zhigang Guo

**Affiliations:** 1 Jiangsu Key Laboratory for Molecular and Medical Biotechnology, College of Life Sciences, Nanjing Normal University, 210097 Nanjing, Jiangsu P.R. China; 2 Key Laboratory for Molecular Biotechnology, College of Life Sciences, Nanjing University, 210093 Nanjing, Jiangsu P.R. China; 3 Department of Neurosurgery, The First Affiliated Hospital of Nanchang University, 330006 Nanchang, R.P. China; 4 Department of Oncology-Pathology, Karolinska Institute, Stockholm, 17176 Sweden

**Keywords:** CNS cancer, Cell growth

## Abstract

Glioblastoma multiforme (GBM) is the most malignant primary brain tumor and has the highest mortality rate among cancers and high resistance to radiation and cytotoxic chemotherapy. Although some targeted therapies can partially inhibit oncogenic mutation-driven proliferation of GBM cells, therapies harnessing synthetic lethality are ‘coincidental’ treatments with high effectiveness in cancers with gene mutations, such as GBM, which frequently exhibits DNA-PKcs mutation. By implementing a highly efficient high-throughput screening (HTS) platform using an in-house-constructed genome-wide human microRNA inhibitor library, we demonstrated that miR-1193 inhibition sensitized GBM tumor cells with DNA-PKcs deficiency. Furthermore, we found that miR-1193 directly targets YY1AP1, leading to subsequent inhibition of FEN1, an important factor in DNA damage repair. Inhibition of miR-1193 resulted in accumulation of DNA double-strand breaks and thus increased genomic instability. RPA-coated ssDNA structures enhanced ATR checkpoint kinase activity, subsequently activating the CHK1/p53/apoptosis axis. These data provide a preclinical theory for the application of miR-1193 inhibition as a potential synthetic lethal approach targeting GBM cancer cells with DNA-PKcs deficiency.

## Introduction

Glioblastoma multiforme (GBM), exhibits highly aggressive invasion, a high mortality rate, and high resistance to radiation and cytotoxic chemotherapy, and thus is the most common malignant primary brain tumor and is not surgically curable^1^. Despite the introduction of modern therapeutic approaches, the median overall survival time of GBM patients is less than 1 year^2^. Similar to normal cells, tumor cells are subject to DNA damage, particularly damage from double-strand breaks (DSBs)^3^. In response to DNA damage, cells activate the DNA damage response (DDR) network, allowing DNA repair through the regulation of cell-cycle progression, DNA damage repair or apoptosis^4^. DNA-dependent protein kinase catalytic subunit (DNA-PKcs) is required for DSB repair via the nonhomologous end joining (NHEJ) pathway, while DNA-PKcs deficiency constitutes one of the most typical features of GBMs^5^. However, the factors that can enhance the survival of DNA-PKcs-deficient GBM cells remain unknown. An alternative DSB repair mechanism, such as homologous recombination (HR) or microhomology-mediated end joining (MMEJ), which is also involved in DSB repair, may compensate for NHEJ pathway deficiency^6,7^. These DSB repair pathways comprise multiple components, such as ataxia-telangiectasia mutated (ATM), ATR, RAD51, BRAC1, and FEN1^3,8,9^. HR requires the function of ATM, but the level of ATM is low in GBM cells due to the high expression of miR-100^10,11^. The term synthetic lethality describes a phenomenon in which simultaneous perturbation of two (or more) functionally complementary genes results in loss of cell viability^12–14^. The application of PARP inhibitors as components in synthetic lethal treatments pioneered a promising clinical therapeutic strategy for breast cancers with mutations in BRCA1 or BRCA2^15^. Moreover, other synthetic lethal interactions have suggested more potential specific anticancer drug targets^16–19^.

MicroRNAs (miRNAs or miRs) are small, highly conserved, noncoding RNA molecules that usually repress gene translation by binding to complementary sequences in the 3′ untranslated regions (3′UTRs) of their target mRNAs^20^. Accumulating evidence indicates that miRNAs play crucial roles in the establishment, progression and recurrence of human cancers^21–24^. In addition, many studies have demonstrated synthetic lethal interactions between miRNAs and specific genes in various cancer cells. For instance, synthetic lethality between the miR-17-92 cluster and the tumor suppressor p53 was documented to suppress retinoblastoma formation;^25^ synthetic lethality between miR-206 and c-Myc via direct inhibition of MAP3K13 was also identified^26^. To date, targeting miRNAs may represent an appealing approach for synthetic lethal targeting of cancer cells with a specific genetic deficiency.

As a structure-specific endonuclease, Flap endonuclease 1 (FEN1) plays vital roles in DSB repair via HR and MMEJ, as well as through the long patch base excision repair (LP-BER) pathway^19,27,28^. A higher level of FEN1 expression was detected in rapidly dividing cells, including various types of cancer cells, than in cells with normal division kinetics and is associated with enhanced malignancy and decreased survival rates^29–31^. In addition, robust evidence revealed that silencing FEN1 leads to enhanced cisplatin sensitivity in glioma^32^, indicating a potential strategy for glioma cancer treatment.

In this study, to identify miRNAs that exhibit synthetic lethality with DNA-PKcs, we implemented an efficient genome-wide high-throughput screening (HTS) method to screen an in-house-constructed antisense oligonucleotide library against 2590 human miRNAs. The miR-1193/FEN1 signaling pathway was identified as a promising synthetic lethal regulator in DNA-PKcs-deficient GBM cells. Moreover, disruption of the DNA-PKcs/miR-1193/FEN1 axis may exert anticancer effects.

## Materials and methods

### Cell lines and transfection

U118MG, M059J, M059K, U251, A549, HepG2, Huh7, RPE-1, 293 T cells, and fibroblasts were purchased from the American Type Culture Collection. Cells free of mycoplasma contamination were maintained in Dulbecco’s modified Eagle’s medium (DMEM) or a 1:1 mixture of DMEM and Ham’s F12 medium supplemented with 2.5 mM L-glutamine, 15 mM HEPES, 0.5 mM sodium pyruvate, 1.2 g/L sodium bicarbonate, 0.05 mM nonessential amino acids and 10% fetal bovine serum. Cells were cultured at 37 °C in a humidified atmosphere containing 5% CO_2_. Cells were seeded in 6-well plates and transfected with anti-miR-1193 (antagomir, antisense oligonucleotide with the reverse complementary sequence of miR-1193), anti-miR-NC (neutral control), shFEN1, siDNA-PKcs, ORF DNA-PKcs, siYY1AP1, and ORF YY1AP1 (purchased from Ruibo, Guangzhou China) by using Lipofectamine 2000 (Invitrogen, USA) according to the manufacturer’s instructions. Cells were harvested on day 4 after transfection for further analyses.

### High-throughput screening (HTS)

Methods similar to those previously described were used^33^. In brief, reverse complimentary sequences of 2590 individual mature human miRNAs were synthesized as a genome-wide miRNA inhibitor library (purchased from Ruibo, Guangzhou, China). U118MG and M059J cells (800 cells per well in 50 µl of medium) were transfected separately with each of the 2590 miRNA antisense oligonucleotides (final concentration, 100 μM) in black polystyrene 384-well flat-bottom microtiter plates (Becton Dickinson, NJ, USA) using a Multidrop™ Combi Reagent Dispenser (Thermo Fisher Scientific, MA, USA). On day 4, cells were fixed with 4% paraformaldehyde solution and stained with 1 mg/ml Hoechst (Sigma–Aldrich, MA, USA) for 15 min at room temperature. Each well was subjected to automated image acquisition for nuclei counting at acumen^®^ Cellista laser scanning imaging cytometer (TTP Labtech, UK).

### Hit selection

Antisense oligonucleotides inhibiting miRNAs were selected as hits based on the nuclei count in the U118MG and M059J cell lines. The standard deviation was obtained for each 384-well plate from the median absolute deviation of the number of nuclei in all treated wells. To calculate the Z score of each miRNA inhibitor, we used the population mean and standard deviation for calculation as z = (x − μ)/σ, where x is the number of nuclei in each individual well while μ and σ are the mean and the standard deviation, respectively, of the total number of nuclei in each plate. A robust significance threshold (Z < −1.5) was applied as the cutoff for selection of cell-killing hits to maximally reduce the possibility of false positive identification, implying a probability of 99.93% that a certain antisense oligonucleotide miRNA inhibitor was a true hit (refer to https://www.dummies.com/education/math/statistics/how-to-use-the-z-table/).

### Antibodies and reagents

The anti-DNA-PKcs antibody was purchased from Thermo Fisher Scientific Inc. (A303-967A, USA; 1:2000 dilution). Antibodies specific for ATR (#2853), phospho-ATR (Thr1989) (#58014), CHK1 (#2360), phospho-CHK1 (Ser317) (#2344), phospho-p53 (Ser20) (#9287), p53 (#9282), and GAPDH (#5174) were purchased from Cell Signaling and 1:1000 diluted for use. Antibodies specific for YY1AP1 (NBP1-81763, 1:1000 dilution), YY1 (NBP1-46218, 1:2000 dilution), and FEN1 (NB100-150, 1:1000 dilution) were purchased from Novus Biologicals (USA). Antibodies specific for caspase-3 (ab32351, 1:2000 dilution), cleaved-caspase-3 (ab32042, 1:500 dilution), and Bax (ab182733, 1:2000 dilution) were purchased from Abcam (USA). DNA-PKcs inhibitor: VX-984 (HY-19939S) and NU-7441 (HY-11006) were purchased from MedChemExpress (USA).

### Luciferase plasmid construction and luciferase assay

We constructed a luciferase-UTR reporter plasmid that contained the YYAP1 3′-UTR region carrying a wild-type or mutant miR-1193 binding site (Fig. 4a). In brief, reverse complimentary oligonucleotides for each selected region containing either a putative or mutated hsa-miR-1193 binding site in the 3′-UTR of YY1AP1 were hybridized to generate double-stranded DNA for insertion into the pMIRReporter^TM^ firefly luciferase vector (Applied Biosystems, CA, USA) at the SacI and HindIII sites. All constructs were confirmed by sequencing. For the luciferase assay, 293 T cells were co-transfected with the appropriate plasmids and 100 nM wild-type or mutant miR-1193 mimic in 48-well plates. Cells were harvested 48 h after transfection, lysed and analyzed using a luciferase assay kit (Promega, Madison, WI, USA) according to the manufacturer’s instructions. β-Galactosidase was used for normalization.

### Quantitative RT-PCR (qRT-PCR)

To measure miR-1193 and YY1AP1 mRNA levels, qRT-PCR was performed in triplicate with TaqMan^®^ Universal PCR Master Mix and a specific TaqMan^®^ MicroRNA assay (Applied Biosystems) in an ABI PRISM® 7000 Sequence Detection System (Applied Biosystems). mRNA expression levels were normalized to the U6 expression level, and relative quantification was performed using the 2^−ΔΔCT^ method. Information on primer sequences is presented in Supplemental Table 1.

### Cell survival assay and cell viability assay

Cell survival was assessed by evaluating the colony-forming ability. In brief, M059J, M059K, and U251 cells were seeded in six-well plates (500 cells per well) after transfection with anti-miR-1193 or anti-miR-NC or treatment with a DNA-PKcs inhibitor (VX-984: 0.1, 0.5, and 1 μM or NU-7441: 0.5, 1, and 2 μM) and were subsequently incubated for 2 weeks to allow colonies to develop. To maximize the effects of the antisense miRNAs, the second transfection was performed one week after the first transfection. Cells were continuously exposed to DNA-PKcs inhibitors for 14 days since the day of first transfection. The medium was replaced every 72 h with medium containing fresh DNA-PKcs inhibitors. Cells were finally fixed with cold methanol, and the colonies were stained with crystal violet (in a 100% methanol solution) for manual counting.

The viability of M059J, M059K and U251 cells was assessed with a Cell Counting Kit-8 (CCK8) kit (Cat#NN517, DOJINDO Laboratories, Japan) according to the manufacturer’s instructions. Cells were seeded in 96-well plates and cultured in a 37 °C incubator for up to 4 days, and the OD at 450 nm was measured. All cell-based assays were performed in at least triplicate.

### Immunofluorescence staining

M059J and M059K cells were cultured in six-well plates and transfected with anti-miR-1193 or anti-miR-NC. After 3 days of incubation, cells were washed with PBS and fixed with 4% formaldehyde. Cells were permeabilized with Triton X-100 (0.05%) for 10 min, blocked with 3% BSA in PBS and then incubated overnight at 4 °C with primary antibodies (anti-53BP1, Abcam, ab175933, 1:200 dilution; anti-RPA, Abcam, ab2175, 1: 200 dilution; and anti-γH2AX, Cell Signaling, #2577, 1:800 dilution). Next, cells were washed and incubated with the corresponding AF488- or AF647-conjugated secondary antibody. Finally, cells were washed with PBST and stained with DAPI for 10 min at RT. Images of the mounted slides were acquired with a Zeiss Axiovert 200 M microscope.

### TUNEL assay

M059J and M059K cells were cultured in six-well plates and transfected with anti-miR-1193 or anti-miR-NC for 4 days as described above. Cells were then washed with PBS, fixed with 4% formaldehyde in PBS for 30 min and washed with PBS again. Triton X-100 (1%) and 3% H_2_O_2_ were applied to the cells for 5 min and 10 min, respectively. Next, cells were washed twice with ice-cold PBS, incubated with TdT labeling solution at 37 °C for 1 h, and gently washed with PBS three times. Finally, cells were incubated with 100 µl staining buffer for 30 min in the dark, washed with PBS and stained with DAPI for imaging.

### Assessment of metaphase spread and nuclear morphology

The chromosome breakage assay was performed as described previously^34^. In brief, M059J and M059K cells were transfected with anti-miR-1193 or anti-miR-NC. After 4 days of culture, cells were treated with 0.5% colchicine for 4 h to induce metaphase arrest, and were incubated with hypotonic solution (0.56% KCl) at room temperature for 30 min and then in a 37 °C water bath for 5 min. Fixation with precooled fixation buffer (ethanol: methanol = 1:3) was repeated three times, and a dropper was used to place cells onto a clean slide. Spread cells were incubated at 55 °C overnight and stained with Giemsa solution (GS-500, Sigma) for image acquisition of aberrant chromosomes with a Zeiss Axiovert 200 M microscope.

### Flow cytometric apoptosis assay

The flow cytometric apoptosis assay was carried out with an Annexin-V FITC/PI staining kit (ab14085, Abcam, UK) according to the manufacturer’s instructions. In brief, 2 × 10^5^ M059K or M059J cells per well were seeded into six-well plates and allowed to attach overnight. Cells were then transfected with anti-miR-1193 or anti-miR-NC and incubated for 96 h in a humidified CO_2_ incubator. Subsequently, cells were trypsinized and centrifuged at 1000 rpm for 5 min. The obtained cell pellet was washed with DPBS and stained with both Annexin-V FITC and PI for 15 min. The stained cells were washed again with DPBS to remove excess dye and were finally resuspended in 300 μl of 1× PBS. Apoptotic events were analyzed, and untreated cells were used as the negative control for gating. A total of 10,000 events were recorded during the experiment. The percentages of live, early apoptotic, late apoptotic, and necrotic cells were analyzed with a BD FACSCalibur device and analyzed with FCS express V3 (BD Biosciences, USA).

### EdU FACS analysis

The S-phase analysis was carried out by flow cytometry (BD FACSCanto™ II, BD Bioscience, USA) using EdU staining assay according to the manufacturer’s instructions. In brief, 2 × 10^5^ M059K or M059J cells per well were seeded into six-well plates and allowed to attach overnight. Cells were then transfected with anti-miR-1193 or anti-miR-NC, fixed with 4% formaldehyde in PBS, washed with 1% BSA and incubated for 30 min with Click-iT EdU reaction solution. After incubation, samples were washed, resuspended in 1% BSA, and analyzed with a BD FACSCalibur device and analyzed with FCS express V3 (BD Biosciences, USA).

### Western blot analysis

Cells subjected to different treatments were harvested and lysed in lysis buffer (50 mM Tris-HCl (pH 7.4), 0.15 M NaCl, and 1% Triton X-100 in PBS, supplemented with protease and phosphatase inhibitors) on ice for 30 min. Proteins were separated by SDS-PAGE on an 8–16% gel (Invitrogen) and transferred to a PVDF membrane. After blocking, the membrane was incubated with primary antibodies (described in the ‘Antibodies and reagents’ sections) and the corresponding secondary antibodies. Immunoreactions were visualized using ECL western blot detection reagents (Pierce Biotechnology) and Image Lab 5.1 gel densitometry analysis system. ImageJ software (version 1.8.0.) was used to analyze protein bands.

### Statistical analysis

All experimental data are expressed as the mean and standard deviation (mean ± SD) values. Statistical analysis was performed using Student’s independent *t*-test, and two-sided *p*-values were calculated via GraphPad Prism 8.4.2 software to assess the significance of differences between experimental groups. A *p*-value of < 0.05 was considered to indicate a significant difference.

## Results

### Identification of miR-1193 as a synthetic lethal partner of DNA-PKcs by high-throughput screening

**Fig. 1:**
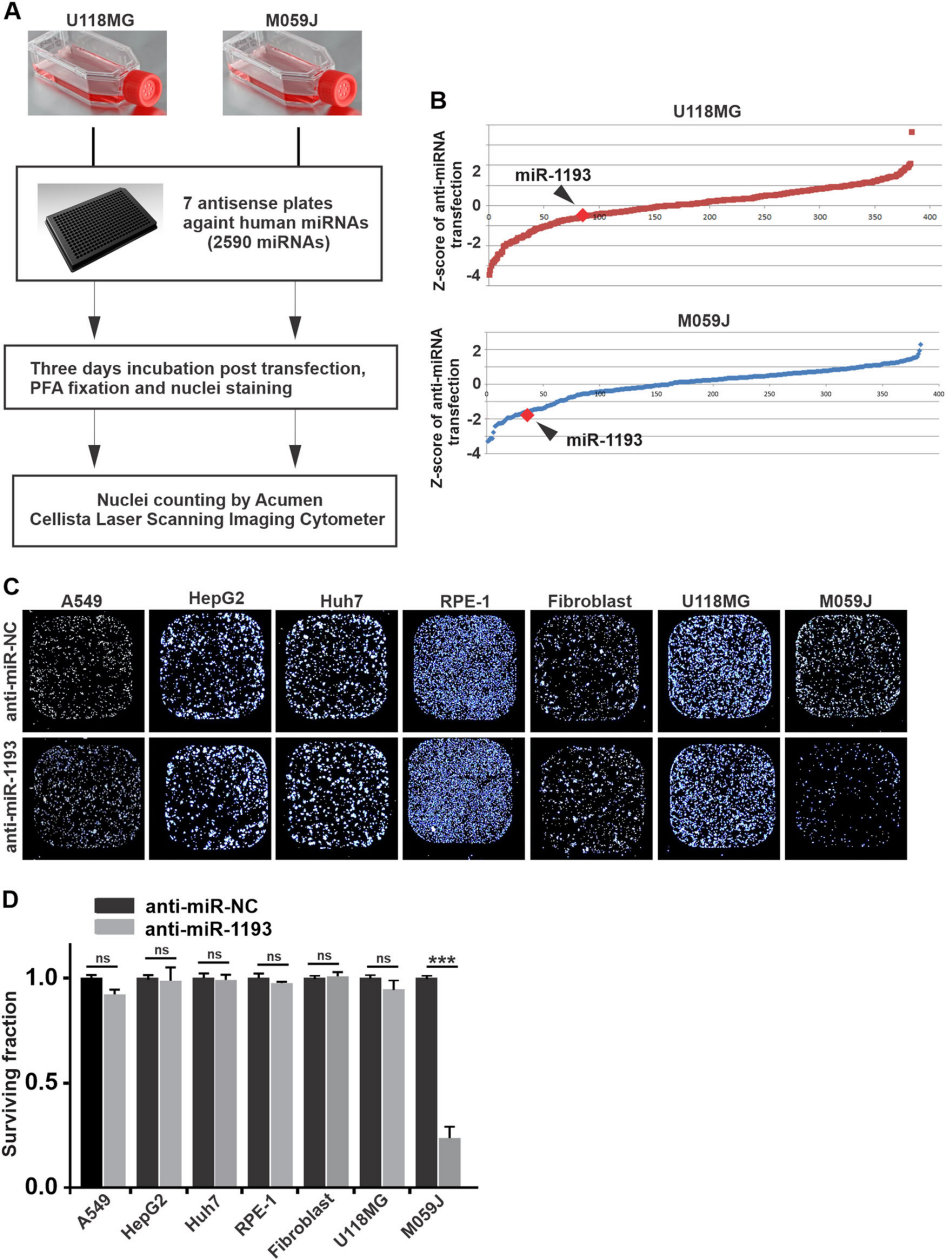
miR-1193 was identified from an automated, high-throughput screen as a specific synthetic lethal interacting partner with DNA-PKcs in M059J cells. **a** Workflow of the anti-miRNA screening procedure. GBM cells (M059J and U118MG) were seeded into seven 384-well plates in 50 μl of medium on the first day. In-house designed antisense oligonucleotides targeting 2590 human miRNAs were individually transfected to knockdown the corresponding miRNAs. Cells were fixed, and nuclei were stained with Hoechst on day 4. Automated nuclei counting was performed with an acumen® Cellista laser scanning imaging cytometer. **b** Z scores were calculated from the screen of all antisense oligonucleotides targeting 2590 human miRNAs in both U118MG cells and DNA-PKcs-deficient M059J cells. The Z scores for miR-1193 are indicated in red and identified by black arrows. **c** Effects of anti-miR-1193 on the proliferation of U118MG, M059J and several other cell lines. **d** The survival fractions were calculated. The data are presented as the mean ± SD values from triplicate biological experiments. *P < 0.05, **P < 0.01, ***P < 0.005. NS not significant: *p* > 0.05.

Many miRNAs are related to cancer cell proliferation, development, and migration. To discover effective miRNA partners with synthetic lethality phenotypes in DNA-PKcs-deficient GBM cells, an automated, miniaturized screening approach was established to minimize human interference and systematic errors. We performed a series of high-throughput screens in seven 384-well plates containing cells from two GBM cell lines—the DNA-PKcs proficient U118MG cell line and the DNA-PKcs-deficient M059J cell line—transfected with a library of 2590 in-house-designed antisense oligonucleotides with reverse complementary sequences of the genome-wide human miRNAs (Fig. 1a). Three days post transfection, the number of nuclei in each well was counted using an acumen^®^ Cellista laser scanning imaging cytometer.

Data were obtained from replicate (*n* = 3) screens for computation of Z scores to estimate the significance of the effect of miRNA knockdown on cell growth. The screen identified miR-1193 as a potential miRNA partner with synthetic lethality in DNA-PKcs-deficient M059J cells (Z score = −1.75) compared with DNA-PKcs-proficient U118MG cells (Z score = −0.3) (Fig. 1b). To validate the specificity of the synthetic lethal interaction between anti-miR-1193 and DNA-PKcs deficiency, anti-miR-1193 was transfected into several different human cell lines, including hepatocarcinoma cell lines (HepG2 and Huh7), a lung cancer cell line (A549), a noncancerous cell line (epithelial RPE-1) and fibroblasts. Imaging for nuclei counting (Fig. 1c) and survival assays (Fig. 1d) revealed the sensitivity to anti-miR-1193 transfection in M059J cells but not in U118MG or the other cell lines tested in this study.

### Validation of miR-1193/DNA-PKcs synthetic lethality

**Fig. 2:**
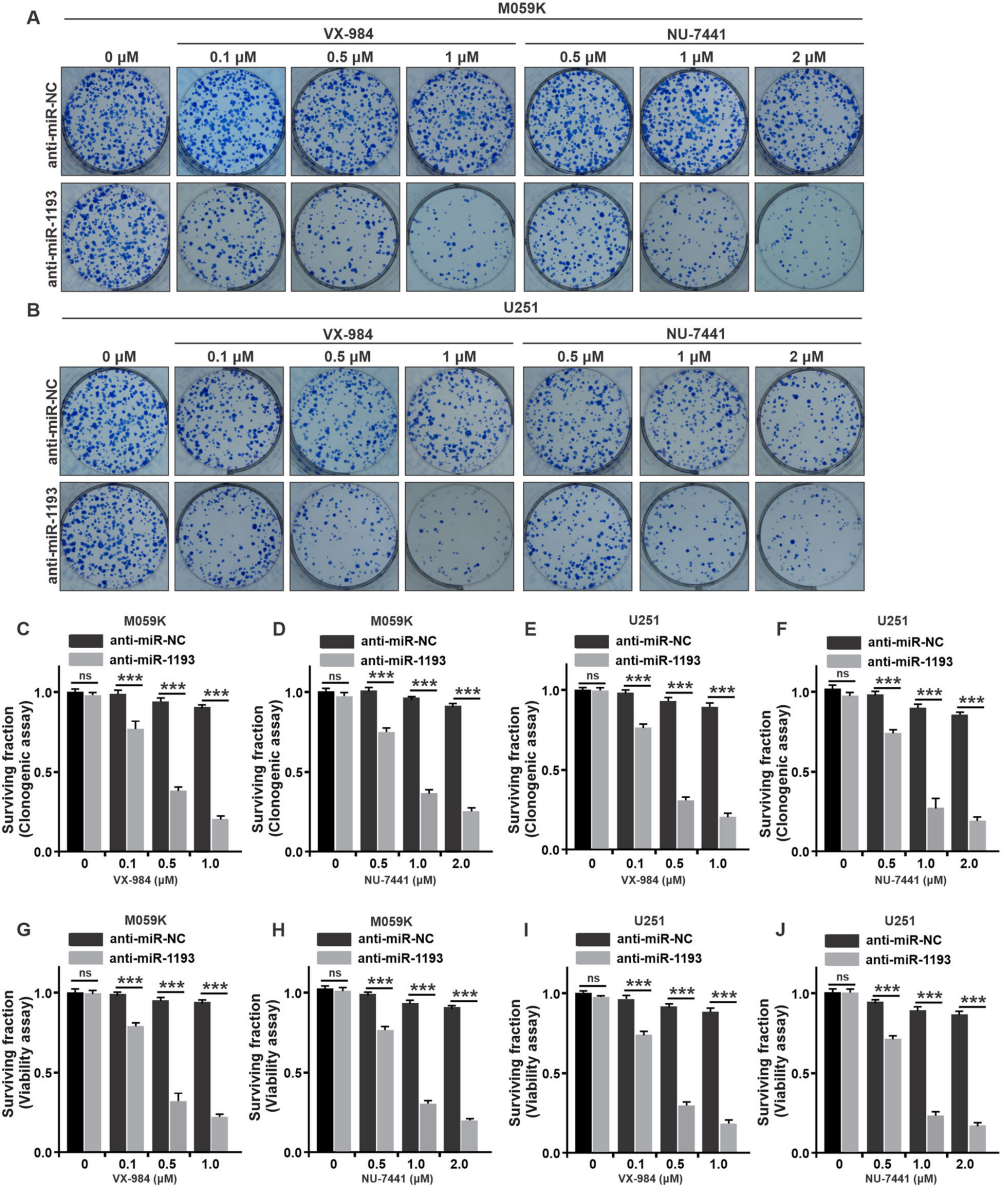
Synthetic lethality of miR-1193 in GBM cells treated with DNA-PKcs inhibitors. Images of colony formation by M059K (**a**) and U251 cells (**b**) treated with the DNA-PK-inhibitors VX-984 (left panel; 0, 0.1, 0.5, and 1 µM) and NU-7441 (right panel; 0, 0.5, 1, and 2 µM) and transfected with anti-miR-1193 or anti-miR-NC. **c**–**f** Survival fractions were calculated. **g**–**j** Cell viability was measured by a CCK8 assay after 4 days of culture. The data are presented as the mean ± SD values from triplicate biological experiments. **P < 0.01, ***P < 0.005. NS not significant: *p* > 0.05.

To further demonstrate the specific synthetic lethal interaction in GBM cells, we treated M059K and U251 cells with two highly specific DNA-PKcs inhibitors, VX-984^35^, and NU-7441^36^, upon the transfection with anti-miR-1193. Similarly, as shown by the results of the clonogenic survival (Fig. 2a–f) and viability assays (Fig. 2g–j), cell proliferation and cell survival were suppressed in a dose-dependent manner when both DNA-PKcs and miR-1193 were inhibited. Collectively, these data suggest that anti-miR-1193 is a specific synthetic lethal partner sensitive to GBM cells deficient in DNA-PKcs.

**Fig. 3:**
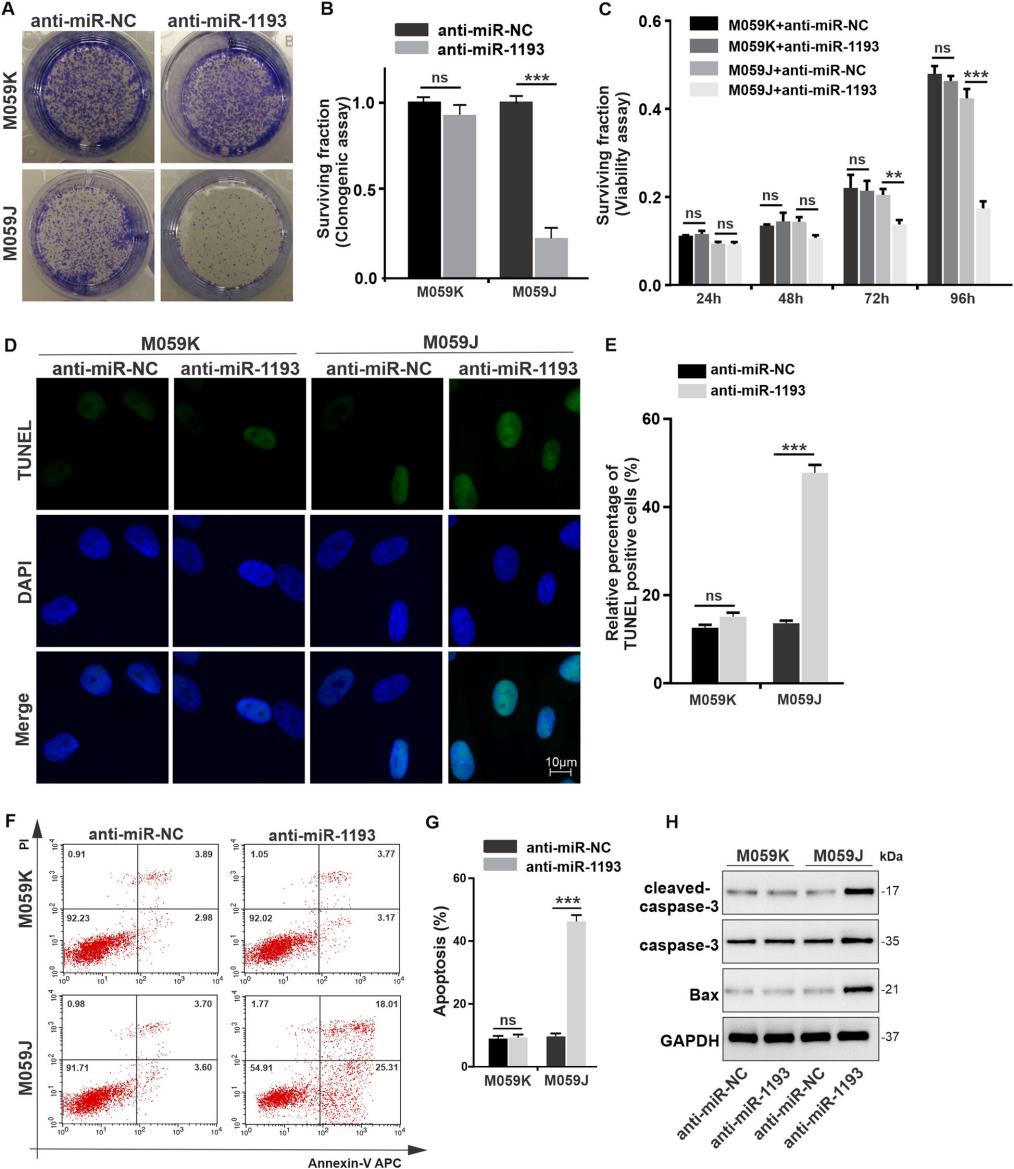
Synthetic lethality between miR-1193 and DNA-PKcs in DNA-PKcs-deficient M059J cells. Isogenic M059J (DNA-PKcs −/−) and M059K (DNA-PKcs+/+) cells were cultured for 4 days after transfection with antisense oligonucleotides targeting miR-1193 (anti-miR-1193) or nontargeting antisense oligonucleotides (anti-miR-NC). **a** Image of colonies from the clonogenic assay in six-well plates. **b** Bar plots of clonogenic survival data. **c** Time course of cell viability using the CCK8 reagent kit. **d** Assessment of apoptosis by a TUNEL assay. **e** Quantification of the apoptotic fraction from **d**. **f** Assessment of apoptosis by an Annexin-V/PI assay. **g** Quantification of the apoptotic fraction from **f**. **h** Cell death proteins were assayed by western blotting. Images and blots are representative of three independent biological replicates. The data are presented as the mean ± SD values, and the error bars represent data from triplicate biological experiments. *P < 0.05, **P < 0.01, ***P < 0.005. NS not significant: *p* > 0.05.

We also confirmed the synthetic lethality of anti-miR-1193 treatment/DNA-PKcs deficiency using some other approaches in M059J and M059K cells with the same origin and thus the same genetic background. The expression levels of miR-1193 and DNA-PKcs in M059J and M059K cells after miR-1193 expression was inhibited by antisense oligonucleotide transfection demonstrated effective inhibition of miR-1193 expression in both cell lines (Fig. S1a) but deficiency of DNA-PKcs only in M059J cells (Fig. S1b). In the clonogenic survival assays, miR-1193 inhibition significantly impaired the survival of DNA-PKcs-deficient M059J cells compared with DNA-PKcs-proficient M059K cells (Fig. 3a, b). In addition, significant synthetic lethal inhibitory effects of anti-miR-1193 in DNA-PKcs-deficient cells were observed 96 h post transfection by a CCK8 cell viability assay (Fig. 3c). The TUNEL assay results demonstrated much more significant apoptotic DNA fragmentation in M059J cells due to knockdown of miR-1193 than in M059J cells (Fig. 3d, e), compared with nontargeting knockdown or in M059K cells with inhibition of miR-1193. Similar results were obtained with Annexin-V/PI staining (Fig. 3f, g). Cell death related proteins were also assayed, with the level of cleaved-caspase-3 and Bax significantly enhanced in M059J cells with miR-1193 deficiency (Fig. 3h). Collectively, our data suggest specific synthetic lethality of miR-1193 inhibition with DNA-PKcs deficiency in M059J cells.

### YY1AP1 is a direct downstream target of miR-1193

Identification of miR-1193 targets is critical for understanding the biological functions of this miRNA in mediating synthetic lethality in M059J cells. Therefore, we compiled two lists of candidate target genes for miR-1193 (data not shown) from two well-recognized prediction databases, i.e., miRDB (version 4.0) and TargetScan (release 6.2) and found that *YY1AP1* was one of the top candidate genes in both lists. Our previous study showed that *YY1* can downregulate *FEN1* expression by directly binding to the *FEN1* coding sequence^37^. *FEN1* plays essential roles in repair of DNA DSB damage and thus promotes tumor cell survival^38^, furthermore, YY1AP1 is related to transcriptional regulation, DNA repair and replication presumably through interacting with YY1^39^. Thus, we focused on *YY1AP1* as the candidate target gene of miR-1193 for further investigations in this study and reasoned that *YY1AP1* might regulate the expression of both *FEN1* and *YY1*. To validate the direct regulation of FEN1 by YY1AP1/YY1, the expression levels of YY1 and FEN1 were measured after YY1AP1 was overexpressed or ablated in 293 T cells. FEN1 expression was clearly suppressed but YY1 expression was enhanced by transfection of the YY1AP1 expression plasmid (ORF YY1AP1). On the other hand, when YY1AP1 was downregulated by YY1AP1 siRNA (siYY1AP1), FEN1 expression was enhanced, but YY1 expression was suppressed. Taken together, these results indicate that as a YY1-associated protein, YY1AP1 can negatively regulate FEN1 expression by interacting with YY1 (Fig. S2a).

**Fig. 4:**
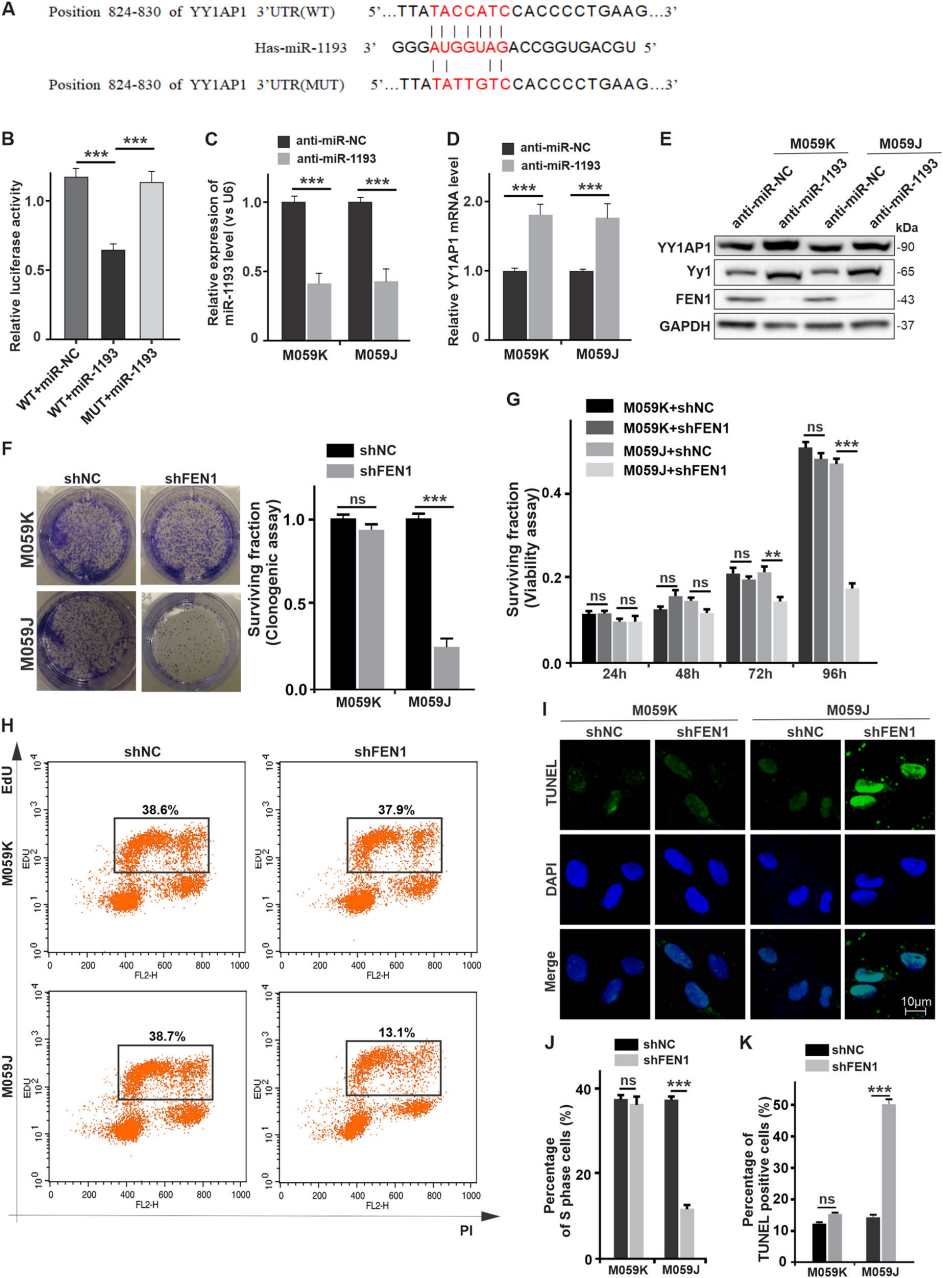
miR-1193 directly targets YY1AP1 and exhibits synthetic lethality with DNA-PKcs through the YY1-FEN1 pathway. **a** Predicted miR-1193 binding sequence in the 3′-UTR of YY1AP1 (wild-type YY1AP1-3′-UTR) and in a mutant containing seven altered nucleotides indicated in red (YY1AP1-3′-UTR-mut). **b** Luciferase activity of the reporter constructs containing the 3′UTR of YY1AP1 with the wild-type or mutated miR-1193 binding site in 293 T cells after co-transfection with the control or miR-1193 expression constructs. The mRNA levels of miR-1193 (**c**) and YY1AP1 (**d**) were measured by RT-qPCR in M059K or M059J cells transfected with anti-miR-NC or anti-miR-1193. U6 RNA was used as the internal control. **e** Protein expression levels of YY1AP1, YY1, and FEN1 in M059K and M059J cells transfected with anti-miR-NC or anti-miR-1193. GAPDH was used as the loading control. **f** Colony formation assay of M059K and M059J cells transfected with shNC and shFEN1 and cultured for 4 days. The expression levels of FEN1 and GAPDH are presented. **g** The viability of transfected M059K and M059J cells was measured with a CCK8 kit across a time course. **h** Cell proliferation was measured by an EdU analysis of FACS. **i** Apoptosis was measured by a TUNEL assay and quantified. **j**, **k** The percentage of cells in S-phase and apoptosis under each condition was quantified. The images are representative of three independent biological replicates. The data are presented as the mean ± SD values, and the error bars represent data from triplicate biological experiments. *P < 0.05, **P < 0.01, ***P < 0.005. NS not significant: *p* > 0.05.

Moreover, we found that the 3′-UTR of *YY1AP1* contains a precise miR-1193 binding site (Fig. 4a). We sought to verify that *YY1AP1* is a direct target gene of miR-1193. To this end, we conducted a luciferase reporter assay to determine whether the putative binding site of miR-1193 in the 3’-UTR of *YY1AP1* is required for miR-1193-regulated gene translation. MiR-1193 repressed the activity of the luciferase reporter containing the wild-type *YY1AP1*-3′-UTR but barely affected the activity of the reporter containing the mutated miR-1193 binding site (Fig. 4b). Furthermore, *YY1AP1* mRNA expression was significantly downregulated (Fig. 4d) upon anti-miR-1193 transfection (Fig. 4c) in both M059J and M059K cells.

### DNA-PKcs-deficient cells are more sensitized to anti-miR-1193 treatment through the YY1AP1/YY1/FEN1 pathway

To examine the effect of anti-miR-1193 on the YY1AP1/YY1/FEN1 axis in DNA-PKcs null cells, we measured the protein expression levels of YY1AP1, YY1, and FEN1 after anti-miR-1193 transfection. As expected, compared with anti-miR-NC transfection, anti-miR-1193 transfection increased the endogenous protein levels of YY1AP1 and YY1 in both M059J and M059K cells, while FEN1 expression was abrogated by anti-miR-1193 transfection (Fig. 4e). These results indicate that YY1AP1 expression is directly regulated by miR-1193 via seed-matching sequences.

To evaluate the necessity of FEN1 for GBM cell proliferation, cell survival assays were performed after FEN1 was downregulated by shFEN1 transfection. Significant inhibition of colony formation, cell growth, and cell viability were observed in DNA-PKcs-deficient M059J cells transfected with shFEN1 (Fig. 4f, g). In addition, FEN1 depletion led to decreased proliferation, as assessed by the EdU assay from Fluorescence activated cell scanning (FACS) and immunofluorescence staining (Figs. 4h, S2b, c), and to increased apoptosis, as assessed by the TUNEL assay (Fig. 4i, j), of M059J cells. The evidence presented here reveals that the synthetic lethal interaction between miR-1193 inhibition and DNA-PKcs deficiency significantly suppresses cell growth and promotes apoptosis through the YY1AP1/YY1/FEN1 pathway in M059J cancer cells.

To further confirm the synthetic lethal interaction between anti-miR-1193 and DNA-PKcs deficiency, we depleted both miR-1193 and DNA-PKcs in M059K cells by simultaneous transfection of anti-miR-1193 and DNA-PKcs-targeted siRNA. The results of the clonogenic survival assay (Fig. S3a) showed that, similar to the effects in M059J cells transfected with anti-miR-1193, co-depletion of miR-1193 and DNA-PKcs significantly inhibited the proliferation of M059K cells. Interestingly, when DNA-PKcs was expressed in M059J cells by transfecting the overexpression plasmid ORF DNA-PKcs, the miR-1193 deficiency-induced inhibition of proliferation was reversed (Fig. S3b–e). The TUNEL assay demonstrated a significant enhancement in apoptosis in M059K cells transfected with siDNA-PKcs and a reduction in apoptosis in M059J cells co-transfected with anti-miR-1193 and ORF DNA-PKcs (Fig. S3f, S3g). These data further confirm the synthetic lethal interaction between anti-miR-1193 and DNA-PKcs deficiency.

### Anti-miR-1193 increases DSB damage in M059J cells with DNA-PKcs deficiency

**Fig. 5:**
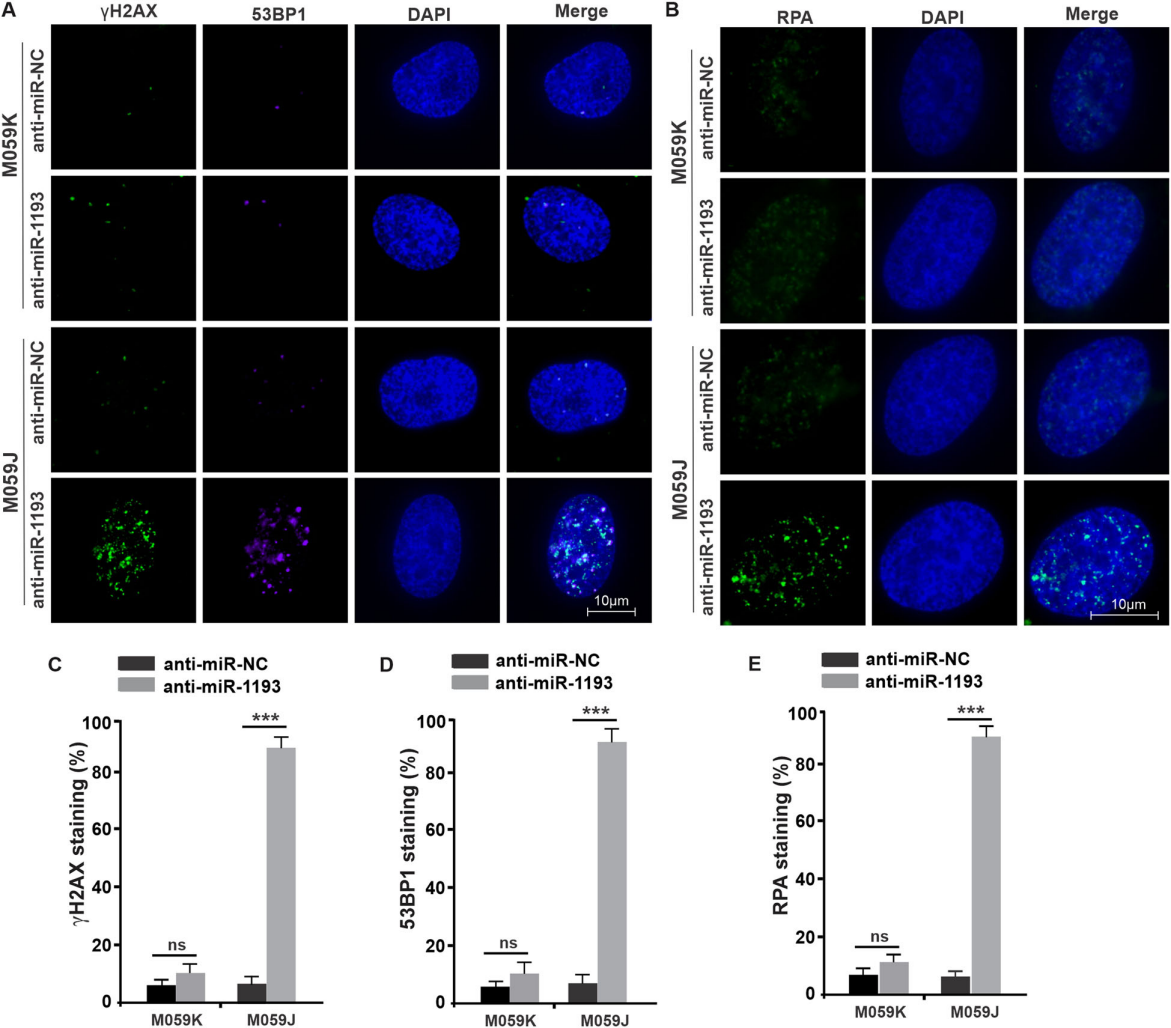
miR-1193 inhibition in DNA-PKcs-deficient cells (M059J cells) leads to DSB repair defects and the formation of RPA-coated ssDNA intermediates. DNA-PKcs-proficient M059K cells and anti-miR-NC were used as controls. **a** Treatment of M059J cells with anti-miR-1193 resulted in DSBs, as indicated by nuclear γH2AX and 53BP1 staining. **b** RPA foci were visualized by immunofluorescence. **c**–**e** Quantification of γH2AX, 53BP1, and RPA foci, respectively. Images are representative of three independent biological replicates. The data are presented as the mean ± SD values, and the error bars represent data from triplicate biological experiments. *P < 0.05, **P < 0.01, ***P < 0.005.

In addition to participating in the LP-BER pathway^40^, FEN1 is also an important regulator of HR and MMEJ-mediated DSB repair^41,42^, suggesting an alternative mechanism by which the DSB repair pathway promotes the survival of DNA-PKcs-deficient M059J cells. In this scenario, DSBs might not be repaired in DNA-PKcs-deficient cells upon depletion of miR-1193/FEN1. To test this hypothesis, an immunofluorescence technique was applied to monitor the presence of γH2AX and 53BP1 nuclear foci, markers of DNA DSB damage^43^, in M059K cells and M059J cells transfected with anti-miR-1193 or anti-miR-NC. Anti-miR-1193 induced the expression of γH2AX, and 53BP1 expression was more pronounced in DNA-PKcs-deficient M059J cells (Fig. 5a, c, and d). A similar induction of γH2AX and 53BP1 expression was detected in M059J cells transfected with shFEN1 (Fig. S4a, S4b, and S4c). Taken together, these observations suggest a DSB repair defect in GBM cells lacking both miR-1193 and DNA-PKcs activity.

To explore the blockade of DSB repair in miR-1193/FEN1-deficient cells, we investigated the ssDNA repair intermediates generated by DNA end resection during the induction of pre-apoptosis in miR-1193/FEN1-depleted cells. The presence of nuclear RPA foci, a marker of ssDNA repair intermediates^44^, was assessed by an immunofluorescence assay in both M059J and M059K cells transfected with anti-miR-1193 or anti-miR-NC. In contrast to M059K cells, M059J cells transfected with anti-miR-1193 exhibited compelling accumulation of RPA foci (Fig. 5b, e), consistent with the low levels of γH2AX and 53BP1 foci (Fig. 4a). Similar results for RPA foci were observed in M059J cells transfected with shFEN1 (Fig. S4d and S4e). Collectively, these data indicate that DSBs persist in DNA-PKcs-deficient cells and yield RPA-coated ssDNA structures upon miR-1193/FEN1 knockdown, promoting the induction of the preapoptotic pathway.

### miR-1193/DNA-PKcs deficiency activates the ATR/Chk1/p53 apoptosis axis

**Fig. 6:**
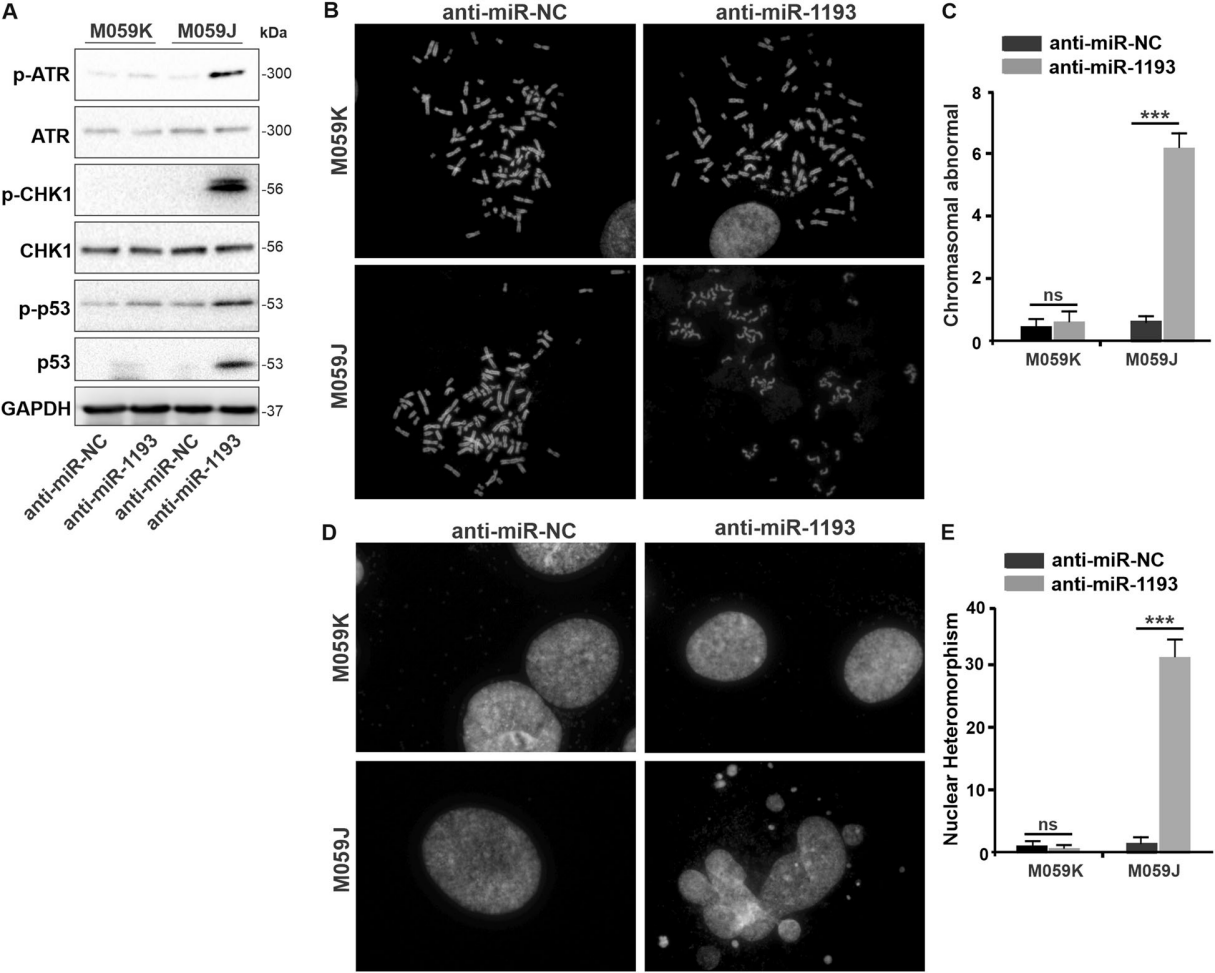
miR-1193 inhibition in DNA-PKcs-deficient cells (M059J cells) results in activation of apoptosis through the ATR/Chk1/p53 axis and promotes chromosomal instability. DNA-PKcs-proficient M059K cells and anti-miR-NC were used as controls. **a** Western blot of p-ATR, ATR, p-CHK1, CHK1, p-p53, p53, and GAPDH. Images of mitotic spread (**b**) and chromosomal abnormalities (**d**) were acquired and quantified. **c**, **e** The images are representative of three independent biological replicates. The data are presented as the mean ± SD values, and the error bars represent data from triplicate biological experiments. *P < 0.05, **P < 0.01, ***P < 0.005. NS not significant: *p* > 0.05.

Subsequently, we sought to determine whether DSB deficiency and ssDNA repair intermediates induce apoptotic signaling in anti-miR-1193-treated/DNA-PKcs-deficient cells. As the expression level of *ATM* is low in M059J cells^10^, we reasoned that the apoptosis induced by anti-miR-1193 treatment/DNA-PKcs deficiency might be dependent on ataxia-telangiectasia mutated and Rad3-related protein kinase (ATR). ATR is a critical component of the cellular DDR, which is activated by DNA damage and replication stress^45^. To test this hypothesis, we assessed the activation status of the ATR/Chk1/p53 signaling axis in M059J and M059K cells transfected with anti-miR-1193 or anti-miR-NC by immunoblotting with antibodies against phospho-ATR (Thr^1989^), phospho-Chk1 (Ser^317^), total p53 and phospho-p53 (Ser^20^). Using these assays, we found the ATR/Chk1/p53 axis to be activated in the DNA-PKcs-defective M059J cells upon miR-1193 inhibition (Fig. 6a). The data in Fig. 6a demonstrate the activation of the ATR/Chk1/p53 apoptosis signaling pathway in miR-1193/DNA-PKcs-deficient M059J cells, suggesting that miR-1193/YY1AP1/YY1/FEN1 inhibition leads to ATR/Chk1-dependent p53 activation in DNA-PKcs-deficient cells.

### Genomic instability is enhanced in M059J cells with miR-1193/DNA-PKcs deficiency

Finally, we observed that inhibition of miR-1193 in DNA-PKcs-deficient M059J cells increased the frequency of chromosomal aberrations compared with that in wild-type M059K cells (Fig. 6b, c). In the nuclear morphology assay, miR-1193 depletion in M059J cells increased the nuclear heteromorphism, with budding cells, micronuclei, and multinucleated giant cells (Fig. 6d, e). Collectively, these data indicated that chromatin instability is significantly increased by accumulated DSB damage and apoptosis activation due to the synthetic lethal interaction between anti-miR-1193 treatment and DNA-PKcs deficiency in GBM cells.

## Discussion

**Fig. 7:**
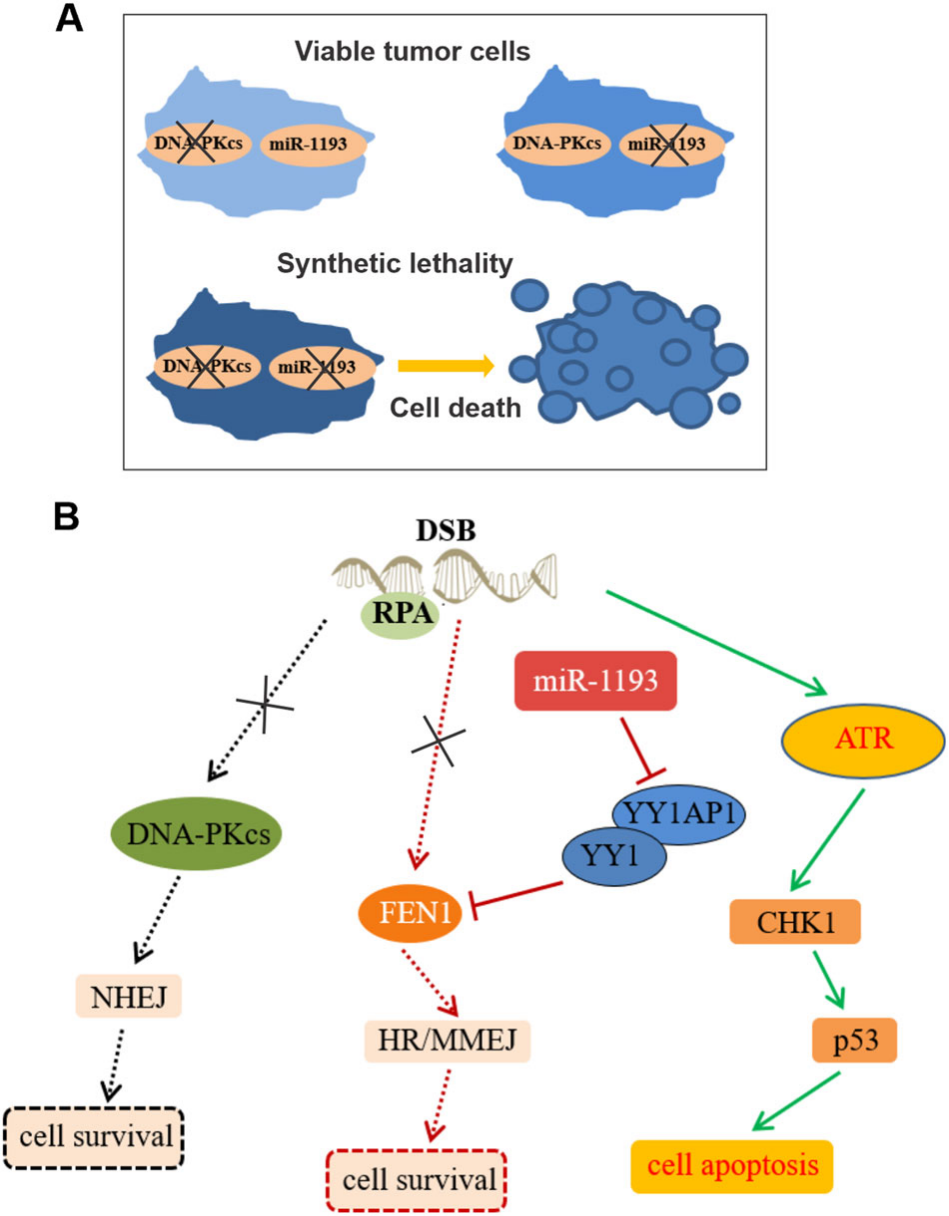
Model of synthetic lethality between miR-1193 inhibition and DNA-PKcs deficiency. **a** Interference with either DNA-PKcs or miR-1193 alone did not reduce the viability of cancer cells, while cell death was achieved by combined abolition of DNA-PKcs and miR-1193. **b** Function loss of DNA-PKcs resulted in dysregulation of the NHEJ-mediated DSB repair pathway (left panel). Inhibition of miR-1193 led to blockade of the FEN1-mediated HR/MMEJ repair pathway (middle panel). Combined abolition of DNA-PKcs and miR-1193 promoted DSB repair deficiency and induced apoptosis regulated by the ATR/CHK1/p53 axis (right panel).

GBM remains the most frequent malignant primary brain tumor, with the highest mortality rate among cancers and very few effective therapies^46^. Synthetic lethality is increasingly recognized as an essential treatment option, especially for cancers harboring certain gene mutations^47^. The flexibility of using miRNA inhibition in synthetic lethal therapies predicts a new era in cancer treatment^25,48^. However, very few synthetic lethal interaction partners have been identified or tested in clinical trials. In this study, we performed a highly efficient automated, high-throughput screen, which identified miR-1193 as a potential anticancer therapeutic candidate for DNA-PKcs-deficient cancers. Based on the robust data shown above, we proposed a working model (Fig. 7) to clarify the mechanism underlying the synthetic lethality between DNA-PKcs deficiency and miR-1193 inhibition. Perturbation of either DNA-PKcs or miR-1193 alone did not decrease the viability of cancer cells (Fig. 7a). The miR-1193/YY1AP1/YY1/FEN1 axis regulates HR and MMEJ-mediated DSB repair, providing a compensating pathway in DNA-PKcs-positive cells in which NHEJ-mediated DSB repair is active. Therefore, perturbation of both DSB repair pathways (HR/MMEJ and NHEJ) by simultaneous inhibition of miR-1193 and ablation of DNA-PKcs can lead to marked loss of viability (Fig. 7b). Simultaneous elimination of these different DSB repair pathways leads to activation of the ATR/CHK1/p53 axis and subsequently promotes tumor cell apoptosis (Fig. 7b).

Our data suggest that anti-miR-1193 could potentially be applied as a single-agent treatment for DNA-PKcs-deficient GBM tumors. The highly malignant nature of human GBMs with DNA-PKcs deficiency and the possibility of inducing synthetic lethality via miR-1193 inhibition provide insights for biomarker-driven animal or clinical trials. In addition to our findings in this study, accumulating data suggest that miRNAs are a valuable means to achieve synthetic lethality in certain tumors with specific genetic deficiencies^49,50^. However, this promising approach still has limitations, among which are potential off-target effects. Chemical modifications of antisense oligonucleotides targeting miRNAs, such as 2′-O-methyl modification, have been shown to minimize the off-target effects and could dramatically increase the in vivo delivery efficiency^51^. A thiol- and cholesterol-conjugated 2′-O-methyl-modified antagomir might be implemented to further validate miR-1193 as a promising therapeutic target for DNA-PKcs-deficient GBM tumors. With the high in vivo delivery efficiency of chemically modified antisense oligonucleotides targeting miRNAs, trials could be conducted in GBM, which exhibits a high frequency of DNA-PKcs deficiency and has a limited standard-of-care therapeutic response and few targeted therapeutic approaches.

In conclusion, this study highlights the importance of the miR-1193/YY1AP1/YY1/FEN1 axis as a promising therapeutic target for DNA-PKcs-deficient GBM tumors, with synthetic lethality as the mechanism of action. The evidence generated in this study provides a novel and promising therapeutic intervention strategy for DNA-PKcs-deficient GBM, and further in-depth investigations are needed.

## Supplementary information

Supplementary Figure Legends

Supplementary Table 1

Text Summary

Supplementary Figure 1

Supplementary Figure 2

Supplementary Figure 3

Supplementary Figure 4
