# Supplementary material for: Inhibition of miR-1193 leads to synthetic lethality in glioblastoma multiforme cells deficient of DNA-PKcs
Source: Cell Death Dis. 2020 Jul 30;11(7):602. doi: 10.1038/s41419-020-02812-3 (PMC7393494; doi:10.1038/s41419-020-02812-3)
Supplement: Supplementary file 1 — Supplementary Figure Legends [file 41419_2020_2812_MOESM1_ESM.docx]

Supplementary Fig. S1. Expression of miR-1193 and DNA-PKcs upon miR-1193 knockdown in M059J and M059K cells. A. RT-qPCR analysis was performed to measure the expression level of miR-1193 in M059J and M059K cells transfected with anti-miR-1193. U6 RNA was used as an internal control. The experiments were performed in triplicate, indicated by the error bars. B. Western blot of DNA-PKcs expression in M059J and M059K cells transfected with anti-miR-1193. The blots are representative of biological triplicate. The data are presented as the mean ± SD values, and the error bars represent data from biological triplicate. *P < 0.05, **P < 0.01, ***P < 0.005. NS, not significant: p>0.05.

Supplementary Fig. S2. The relationship between YY1AP1 and FEN1. A. FEN1 was negatively regulated by YY1AP1. FEN1 level decreased when 293T cells were transfected with ORF YY1AP1 (left panel), while FEN1 level increased when transfected with siYY1AP1 (right panel). B. The proliferation of M059K and M059J cells was measured by EdU immunofluorescence staining and quantified in C. The data are presented as the mean ± SD values, and the error bars represent data from biological triplicate. *P < 0.05, **P < 0.01, ***P < 0.005. NS, not significant: p>0.05.

Supplementary Fig. S3. DNA-PKcs compensation experiments. A. Survival fraction analyzed four days post transfection. M059K cells were transfected with anti-miR-1193 or combined with siDNA-PKcs. M059J cells were transfected with anti-miR-1193 or together with ORF DNA-PKcs. Cell proliferation was either measured by EdU FACS analysis (B) with quantification (C), or by EdU immunofluorescence staining (D) with quantification (E). Cell apoptosis was measured by TUNEL assay (F) with quantification (G). The images are representative of biological triplicate, which were quantified, indicated by the error bars. The data are presented as the mean ± SD. *P < 0.05, **P < 0.01, ***P < 0.005. NS, not significant: p>0.05.

Supplementary Fig. 4. FEN1 inhibition in DNA-PKcs-defective cells (M059J cells) led to DSB repair defect and RPA-coated ssDNA intermediates. DNA-PKcs-proficient M059K cells and shNC were used as control. A. Treatment of M059J cells with shFEN1 resulted in DSBs by nuclear γH2AX and 53BP1 staining, and their quantifications (B and C). D. RPA foci were visualized by immunofluorescence, with its quantification shown in E. The images are representative of biological triplicate, which were quantified, indicated by the error bars. The data are presented as the mean ± SD values. *P < 0.05, **P < 0.01, ***P < 0.005. NS, not significant: p>0.05.
