# Supplementary material for: Inhibition of miR-1193 leads to synthetic lethality in glioblastoma multiforme cells deficient of DNA-PKcs
Source: Cell Death Dis. 2020 Jul 30;11(7):602. doi: 10.1038/s41419-020-02812-3 (PMC7393494; doi:10.1038/s41419-020-02812-3)
Supplement: Supplementary file 2 — Supplementary Table 1 [file 41419_2020_2812_MOESM2_ESM.docx]

**Supplementary Table 1**

| Primer name | sequence |
| --- | --- |
| YY1AP1-F | GAAAACACCAGCCCAATCAAC |
| YY1AP1-R | ACTCAAAACTCTCACCTCCAC |
| WT-YY1AP1-3’UTR-F | ACTAGTGGTTAACCCTACTTCCTTCCC |
| WT-YY1AP1-3’UTR-R | TTCGAAGGGCTCTAATTTGGGTTCTAGG |
| MUT-YY1AP1-3’UTR-F | CTTATCTTCAGGGGTGGACAATATAGGAAGATTCACAGAATTGCCAGAAACAATT |
| MUT-YY1AP1-3’UTR-R | AATTGTTTCTGGCAATTCTGTGAATCTTCCTATATTGTCCACCCCTGAAGATAAG |
