# Supplementary material for: Inhibition of miR-1193 leads to synthetic lethality in glioblastoma multiforme cells deficient of DNA-PKcs
Source: Cell Death Dis. 2020 Jul 30;11(7):602. doi: 10.1038/s41419-020-02812-3 (PMC7393494; doi:10.1038/s41419-020-02812-3)
Supplement: Supplementary file 3 — Text Summary [file 41419_2020_2812_MOESM3_ESM.docx]

**Text Summary**

**Supplementary table 1.docx** contains supplementary table.

**Supplementary figure legends.docx** contains the figure legends for supplementary figures.

**Supplementary Figure 1.tif** contains the Supplementary Figure 1.

**Supplementary Figure 2.tif** contains the Supplementary Figure 2.

**Supplementary Figure 3.tif** contains the Supplementary Figure 3.

**Supplementary Figure 4.tif** contains the Supplementary Figure 4.
