# Supplementary figures and images for: Inhibition of miR-1193 leads to synthetic lethality in glioblastoma multiforme cells deficient of DNA-PKcs
Source: Cell Death Dis. 2020 Jul 30;11(7):602. doi: 10.1038/s41419-020-02812-3 (PMC7393494; doi:10.1038/s41419-020-02812-3)

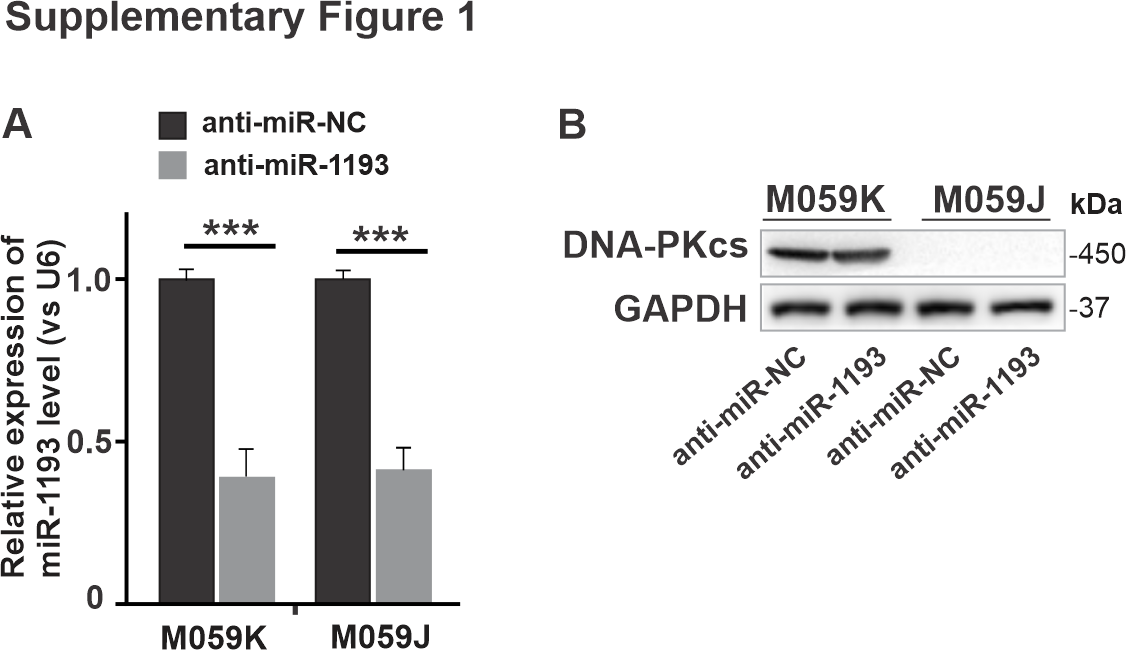

Supplement: Supplementary file 4 — Supplementary Figure 1 [file 41419_2020_2812_MOESM4_ESM.tif]

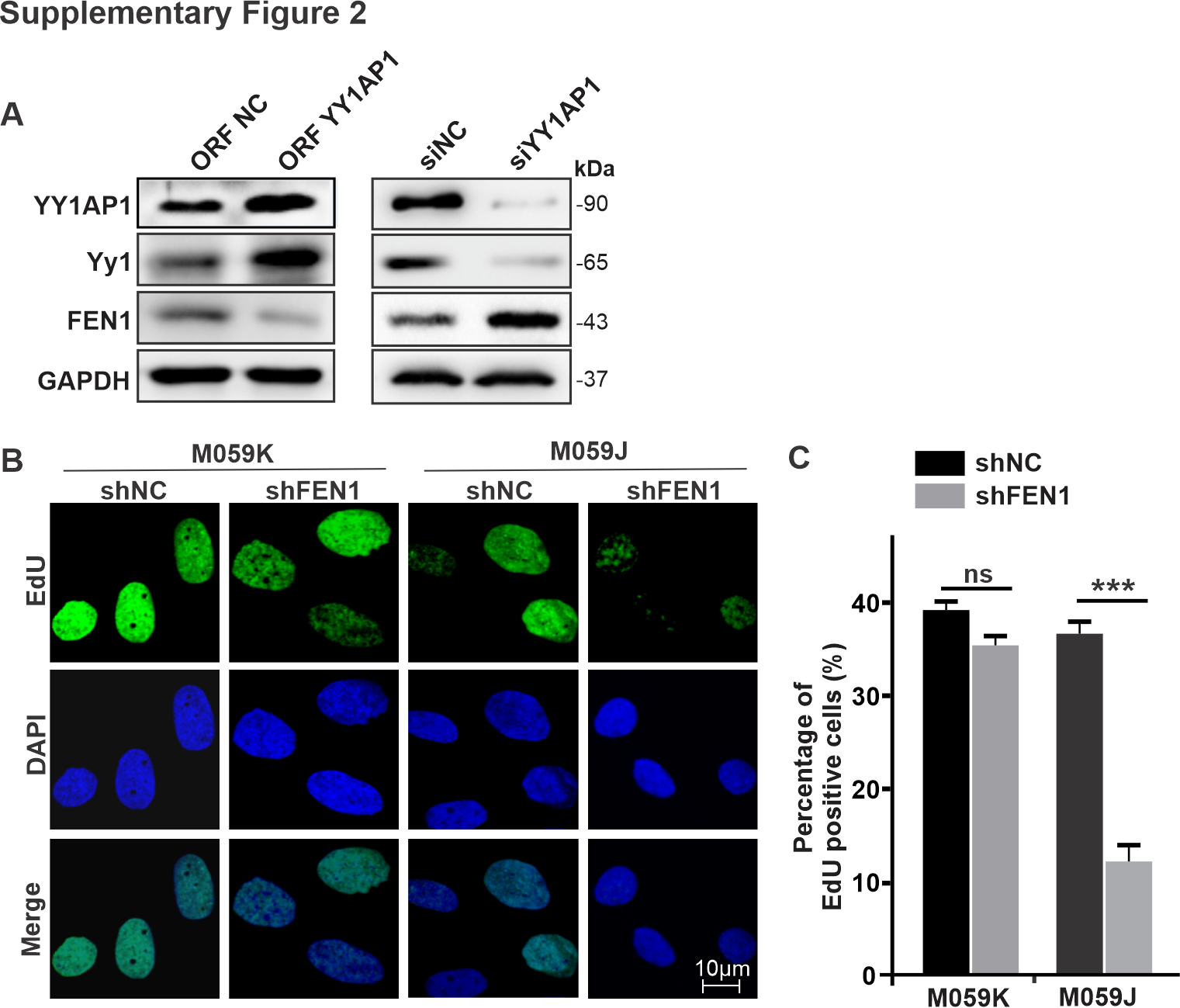

Supplement: Supplementary file 5 — Supplementary Figure 2 [file 41419_2020_2812_MOESM5_ESM.tif]

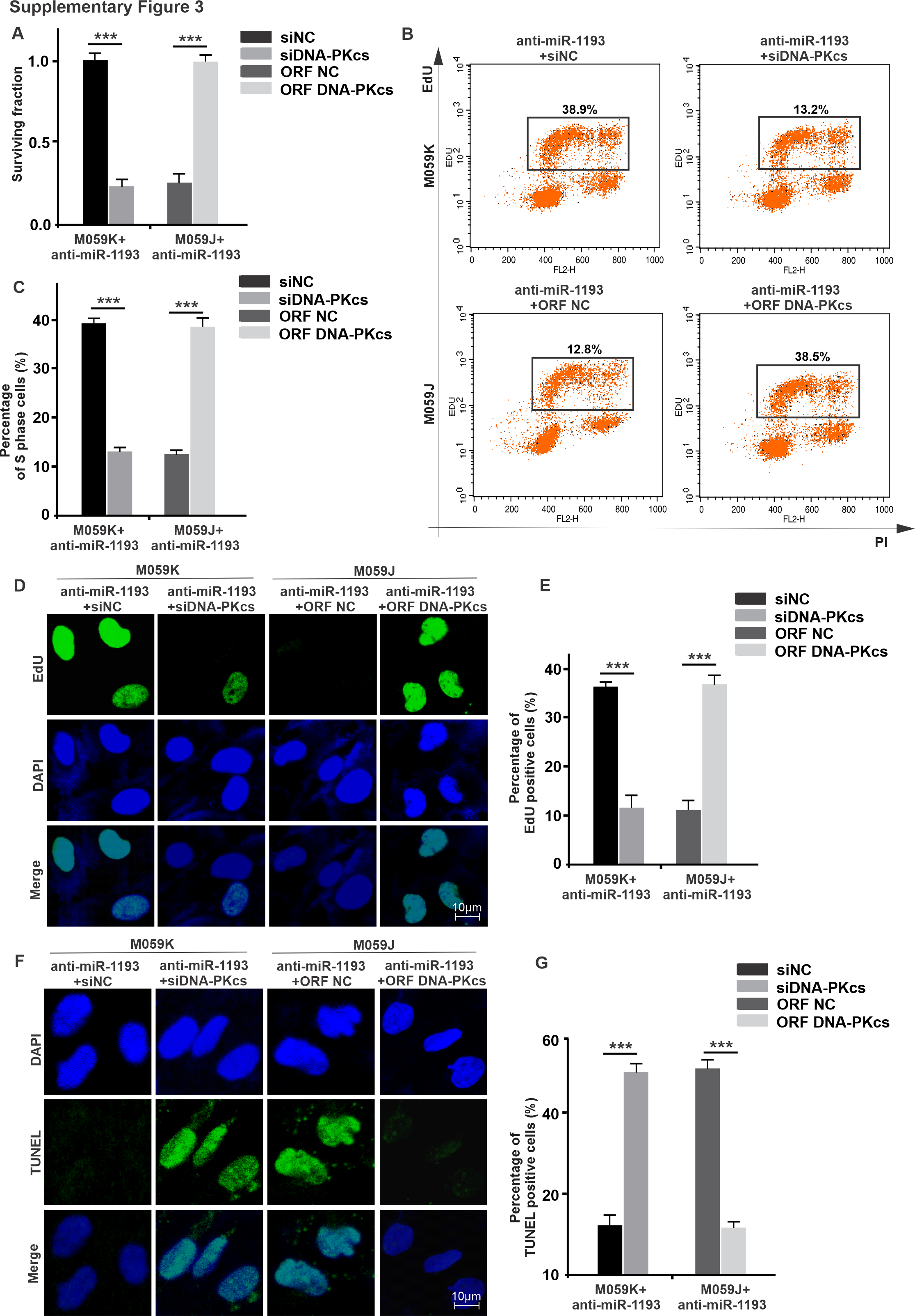

Supplement: Supplementary file 6 — Supplementary Figure 3 [file 41419_2020_2812_MOESM6_ESM.tif]

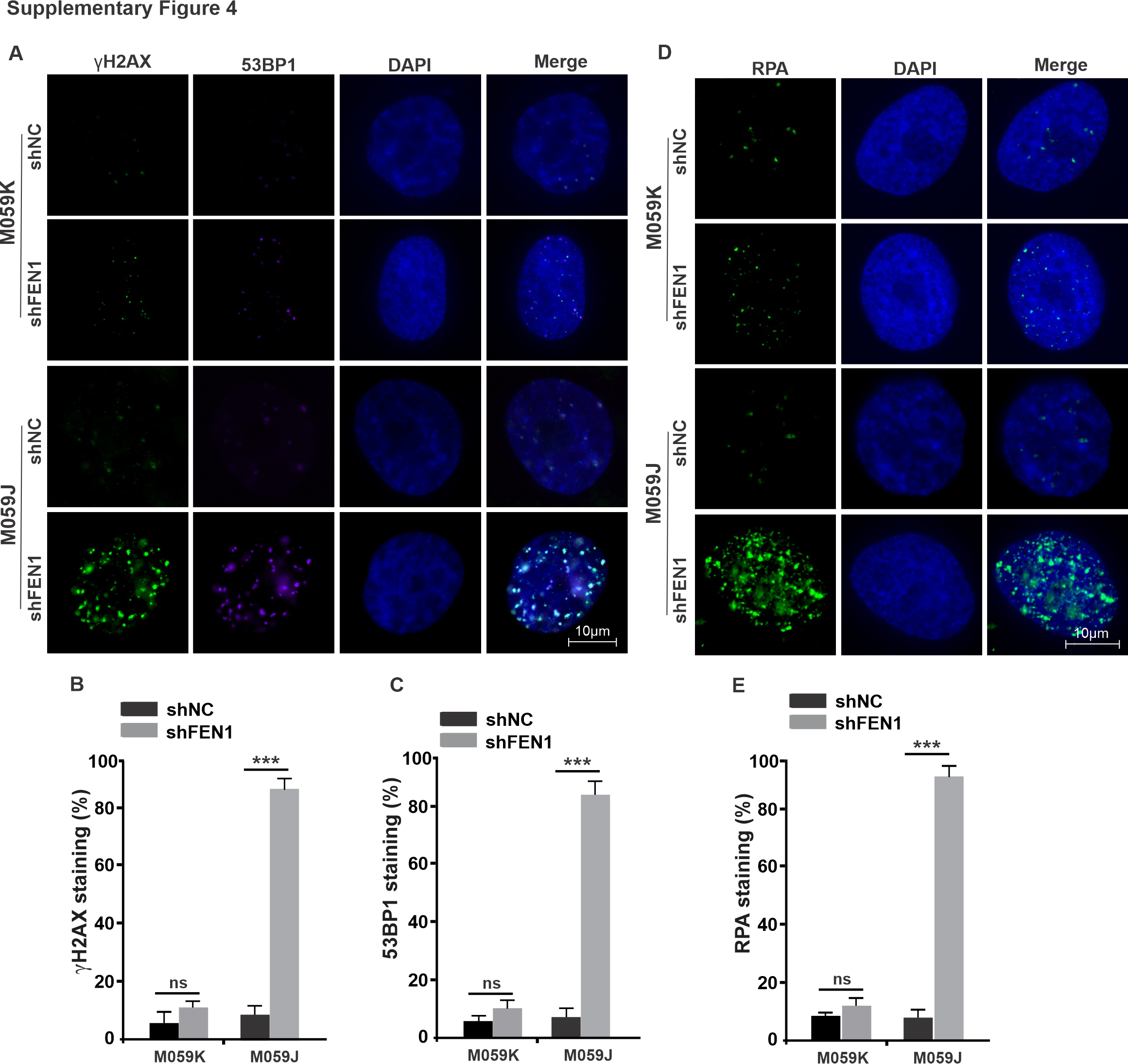

Supplement: Supplementary file 7 — Supplementary Figure 4 [file 41419_2020_2812_MOESM7_ESM.tif]
